# Supplementary figures and images for: β-Catenin-NF-κB-CFTR interactions in cholangiocytes regulate inflammation and fibrosis during ductular reaction
Source: eLife. 2021 Oct 5;10:e71310. doi: 10.7554/eLife.71310 (PMC8555990; doi:10.7554/eLife.71310)

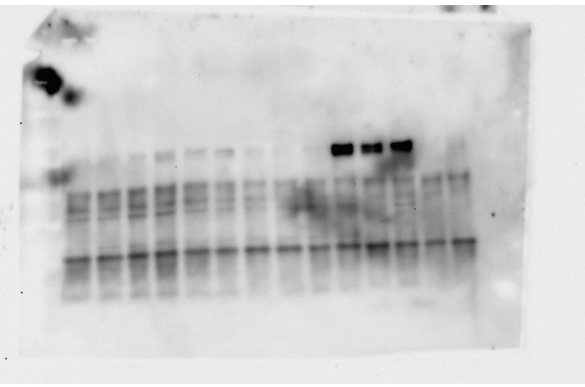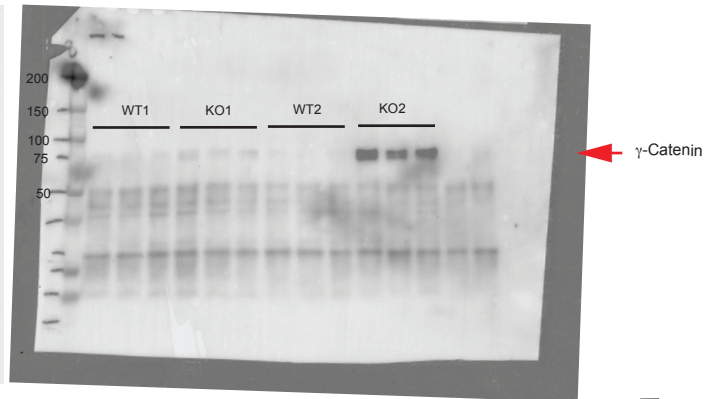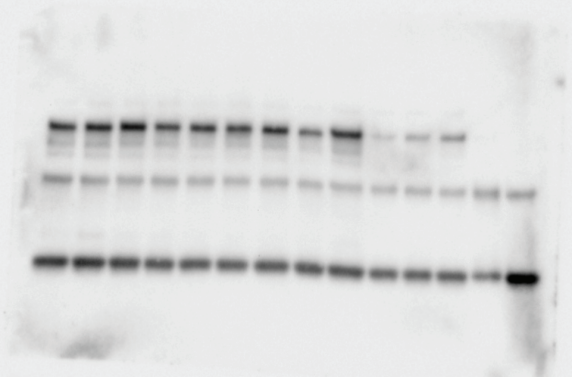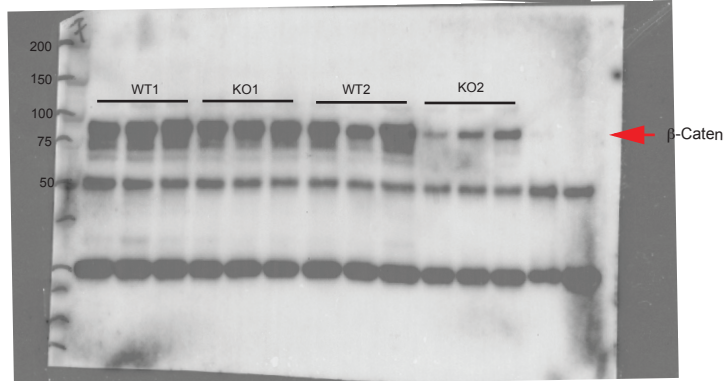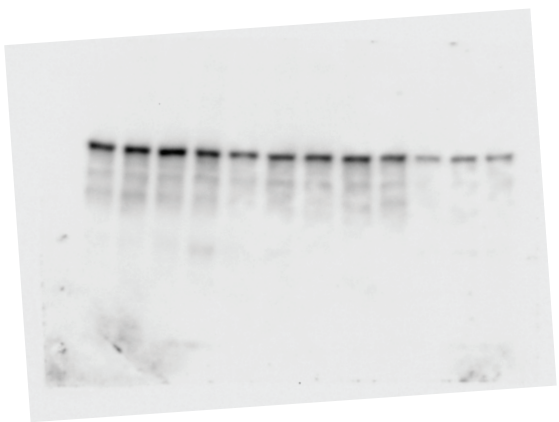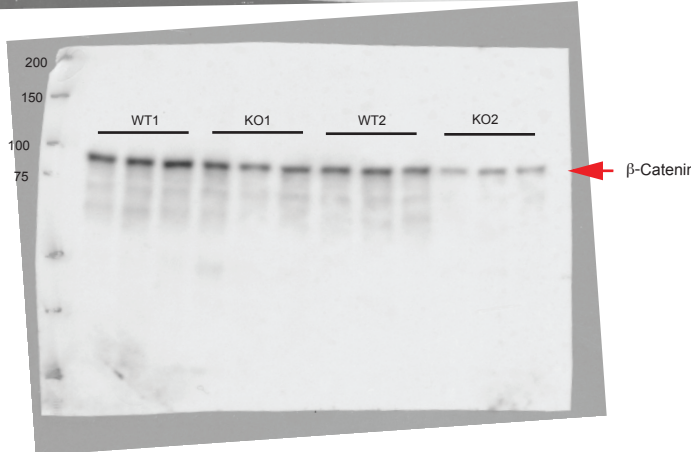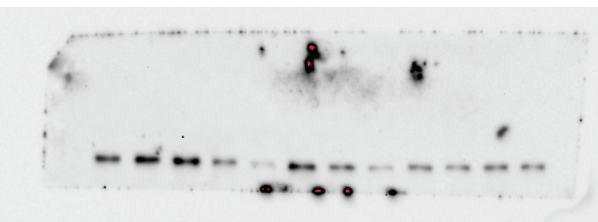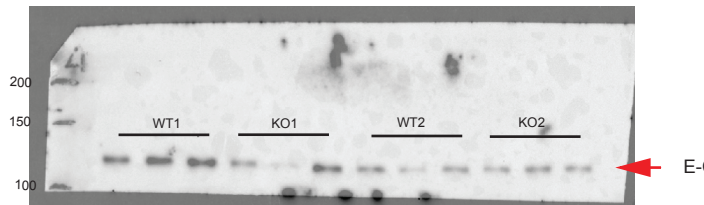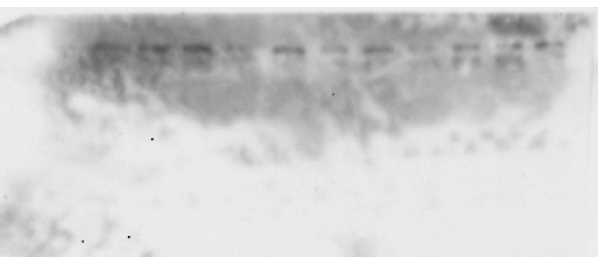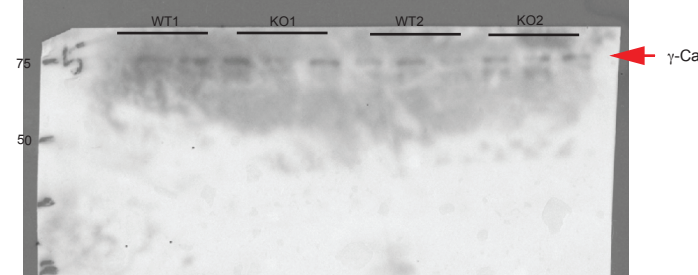

Supplement: Figure 4—figure supplement 3—source data 1. — Input verifies low levels of β-catenin in whole liver lysates of KO1 at the same time depicting β-catenin presence in liver non-epithelial cells (bottom panels). [file elife-71310-fig4-figsupp3-data1.pdf]

Figure 7C

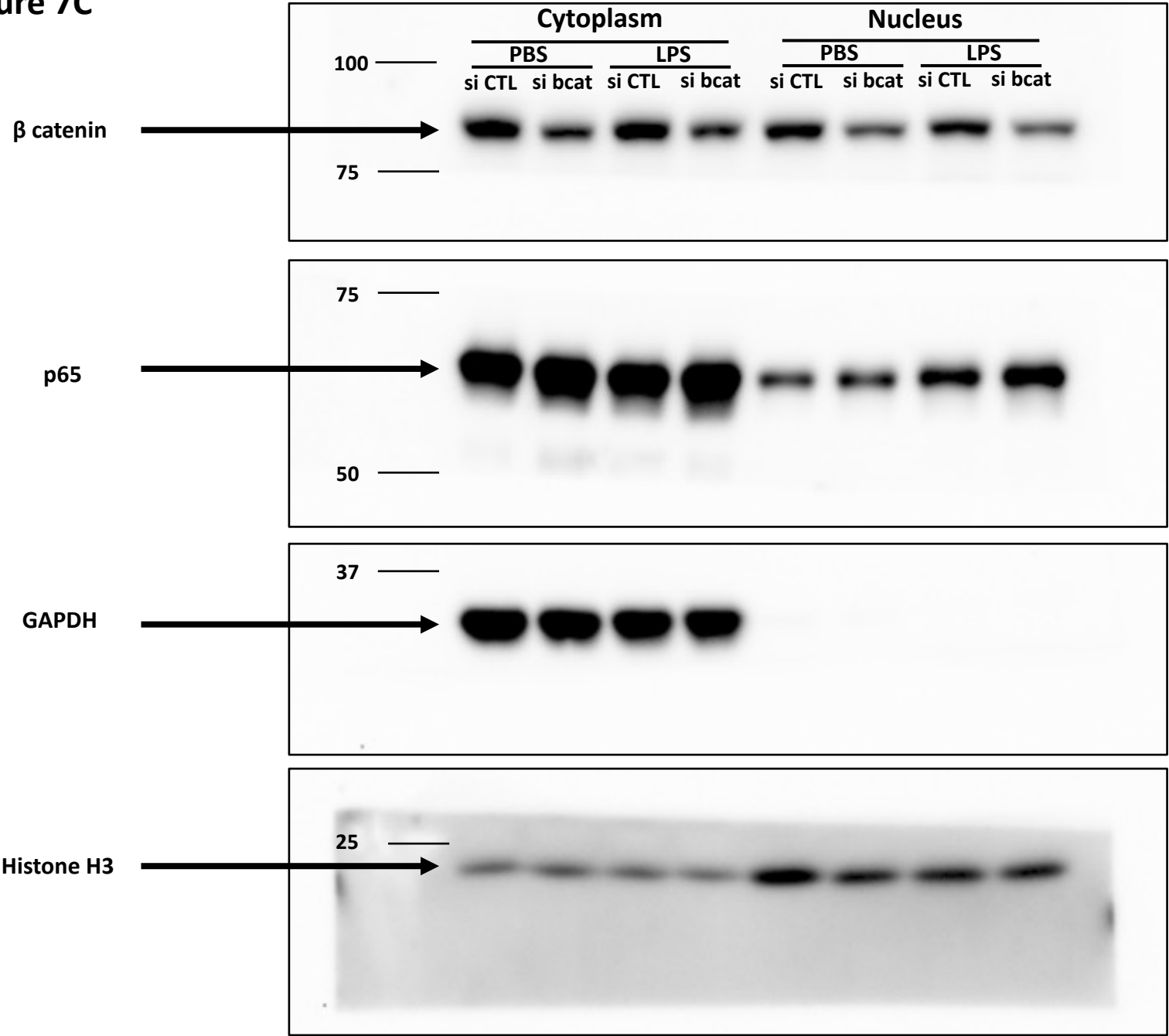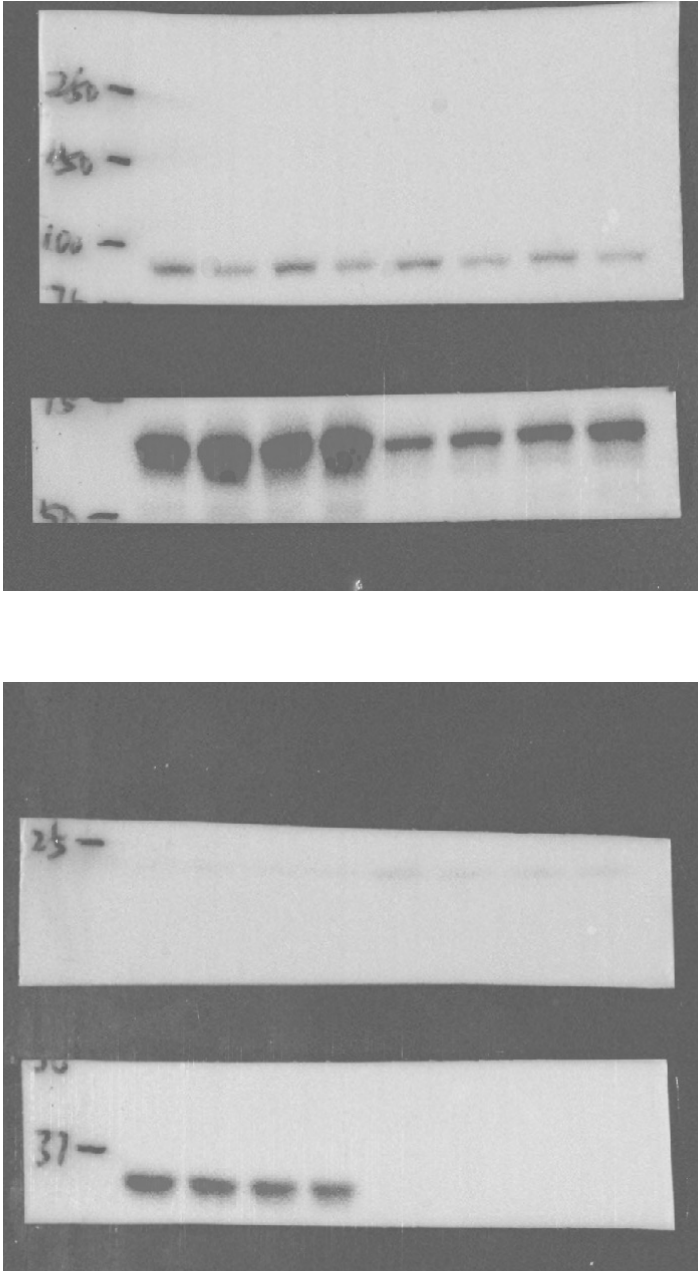

Supplement: Figure 7—source data 1. [file elife-71310-fig7-data1.pdf]

Figure 7H

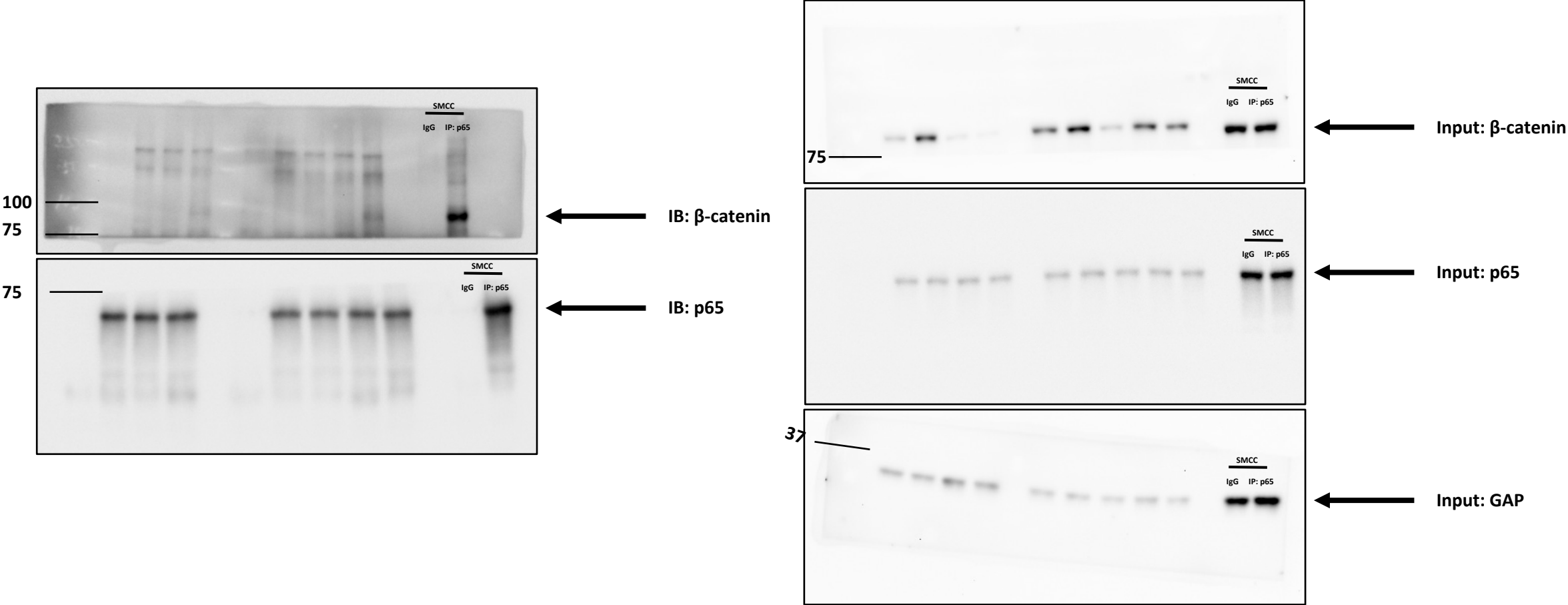

Supplement: Figure 7—source data 2. [file elife-71310-fig7-data2.pdf]

Figure 7I

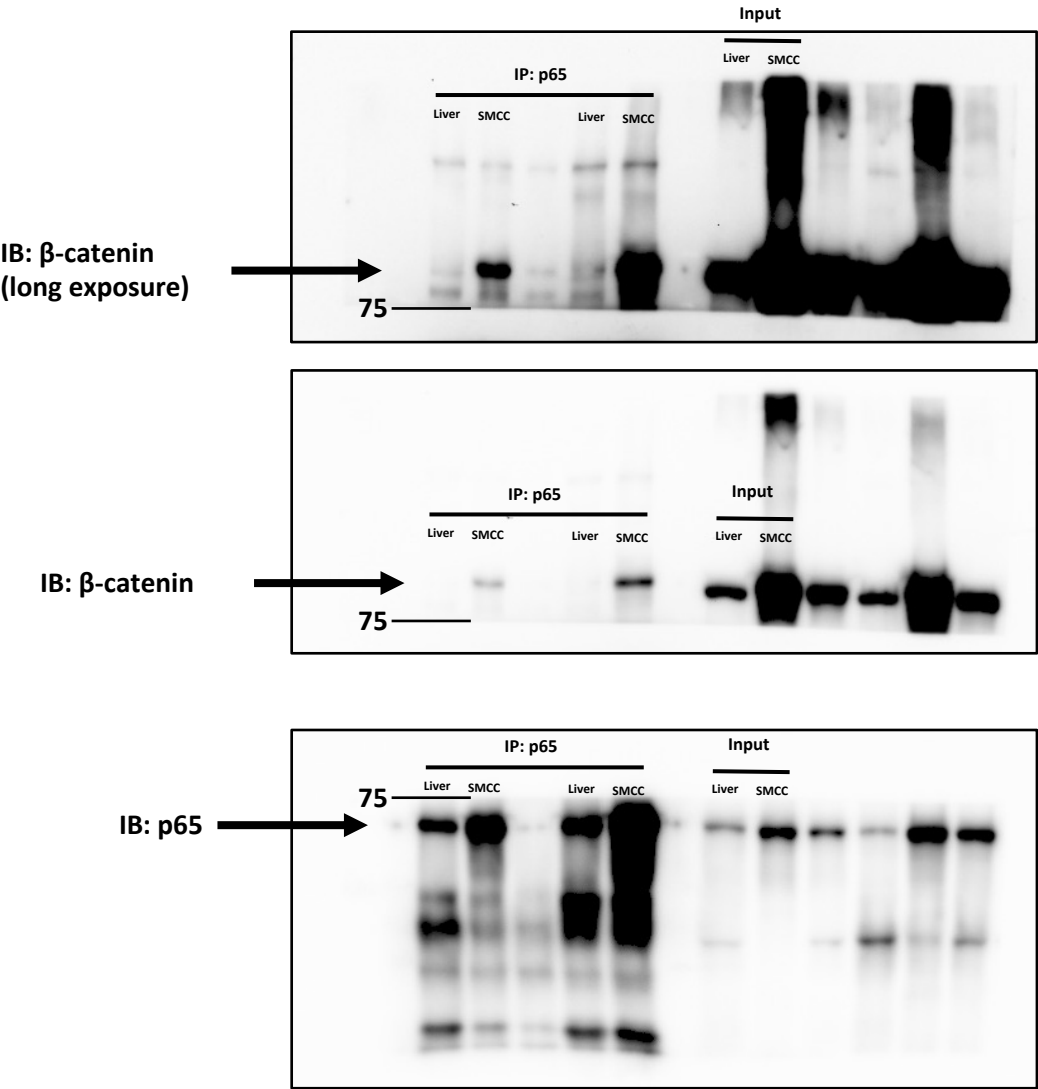

Supplement: Figure 7—source data 3. [file elife-71310-fig7-data3.pdf]

Figure 8C

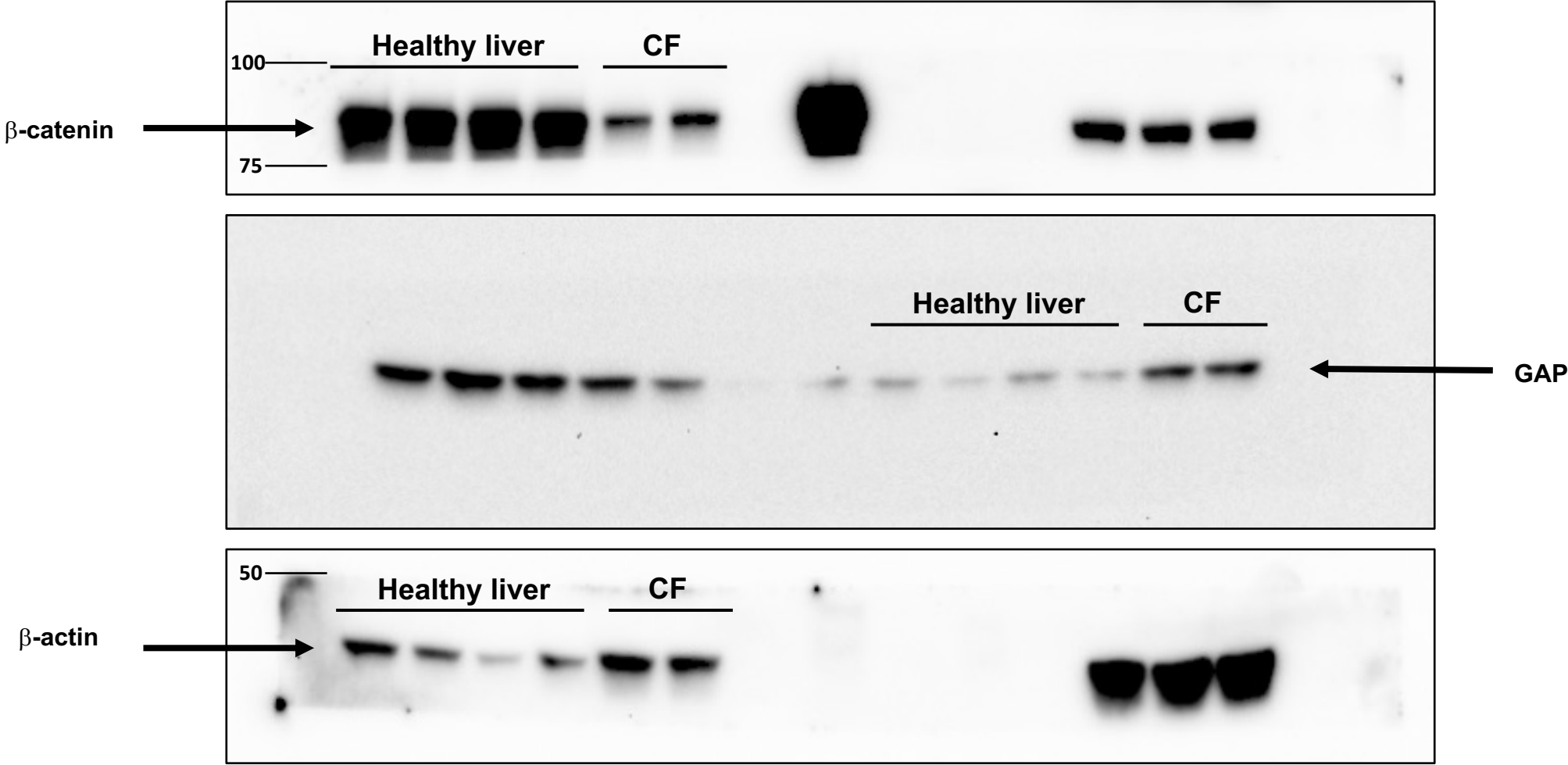

Supplement: Figure 8—source data 1. [file elife-71310-fig8-data1.pdf]

Figure 8D

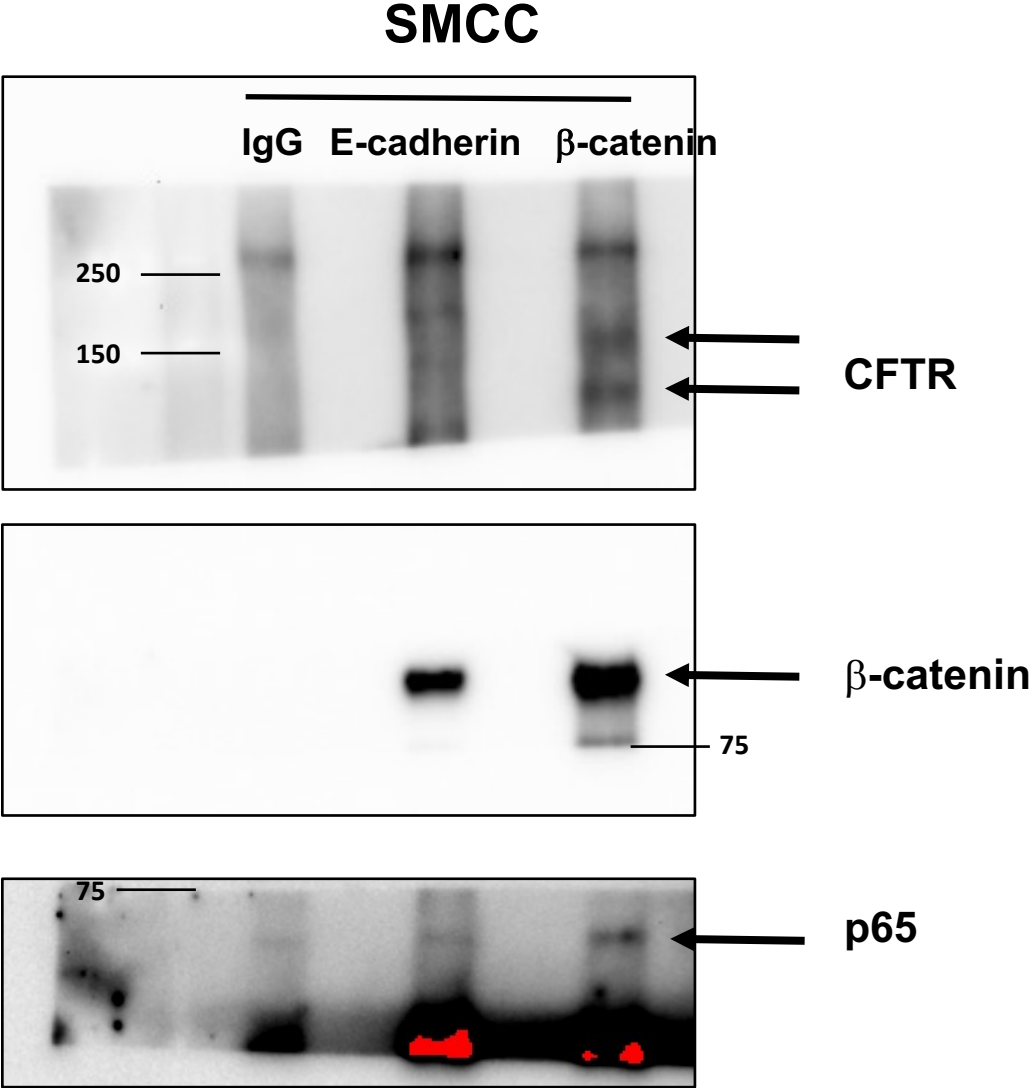

Supplement: Figure 8—source data 2. [file elife-71310-fig8-data2.pdf]

Figure 8E

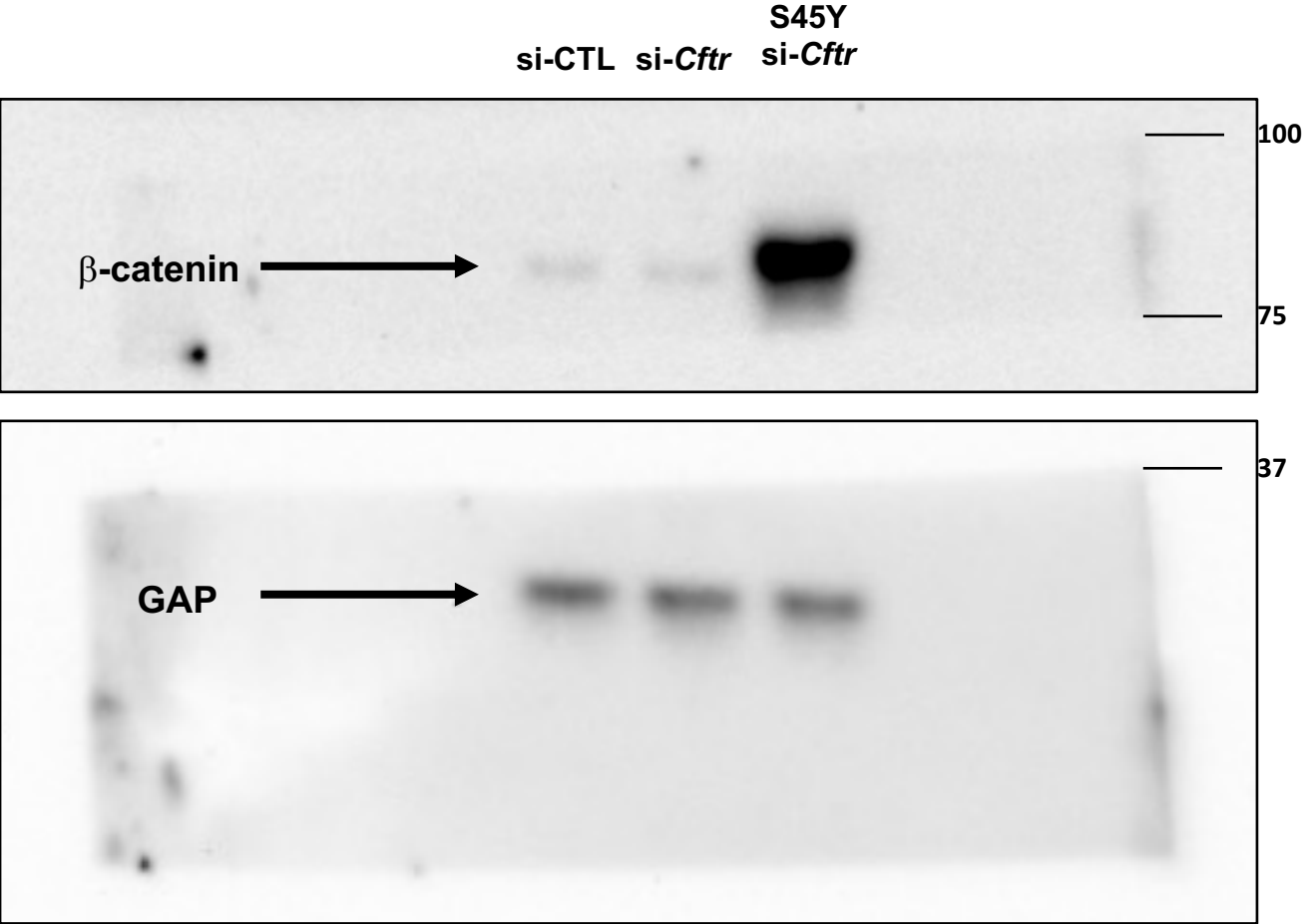

Supplement: Figure 8—source data 3. [file elife-71310-fig8-data3.pdf]

Figure 8-figure supplement 1

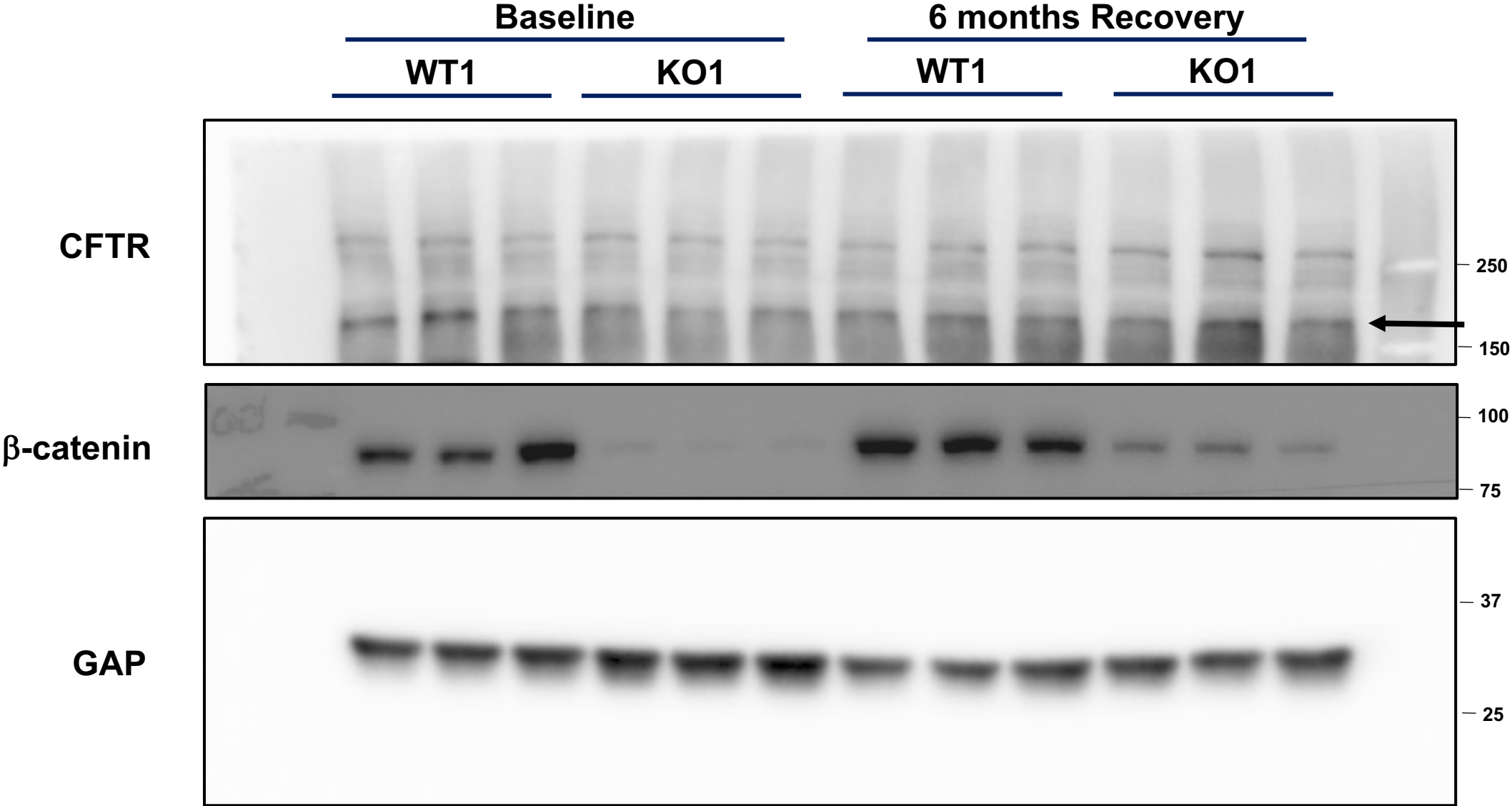

Supplement: Figure 8—figure supplement 1—source data 1. [file elife-71310-fig8-figsupp1-data1.pdf]
