# Supplementary material for: β-Catenin-NF-κB-CFTR interactions in cholangiocytes regulate inflammation and fibrosis during ductular reaction
Source: eLife. 2021 Oct 5;10:e71310. doi: 10.7554/eLife.71310 (PMC8555990; doi:10.7554/eLife.71310)
Supplement: Supplementary file 1. — Patient tissue sections including 5 patients with healthy liver, 12 patients with ductular reaction (DR) from alcoholic hepatitis (AH) (n = 10) and/or NASH (n = 2), 5 patients with DR associated with polycystic liver disease (PLD), and 6 patients with DR in cystic fibrosis (CF) cases were provided by the Pittsburgh Liver Research Center’s (PLRC’s) Clinical Biospecimen Repository and Processing Core (CBPRC). Two pieces of frozen livers from one CF patient (TP10-P531) were provided by Pitt Biospecimen Core. [file elife-71310-supp1.docx]

**Supplementary File 1: Patient samples used in the study**

| **Identifier** | **Age** | **Sex** | **Diagnosis** | **Miscellaneous** |
| --- | --- | --- | --- | --- |
| PHS16-32066 | 66 | M | Non-neoplastic hepatic parenchyma | Mild nodular regenerative hyperplasia and mild portal reactive changes |
| PHS16-40535 | 50 | M | Non-neoplastic hepatic parenchyma | Minimal mixed micro-and macrovesicular steatosis involving approximately 5% of the hepatocytes. Mild chronic portal inflammation. Focal mild portal fibrosis. |
| PHS18-5904 | 71 | M | Background liver | Mild portal inflammation. No significant steatosis or fibrosis |
| PHS18-11592 | 60 | F | Background liver | Moderate macrovesicular steatosis |
| PHS18-34295 | 53 | M | Non-neoplastic hepatic parenchyma | Mild nodular  regenerative hyperplasia |
| PHS18-4910 | 48 | M | Alcoholic hepatitis | Micronodular liver cirrhosis with moderate cholestasis, ductular reaction, abundant Mallory-Denk bodies, neutrophilic lobular inflammation, compatible with clinical history of acute alcoholic hepatitis. Chronic active steatohepatitis (Nash score: 5/8), 15% mixed micro-and macrovesicular steatosis. |
| PHS18-12144 | 72 | M | Alcoholic hepatitis | Active mixed cirrhosis secondary to chronic hepatitis, viral-type C, mildly active. Marked cholestasis and focal parenchymal extinction with prominent ductular reaction. |
| PHS18-42469 | 41 | M | Alcoholic hepatitis | micronodular cirrhosis with extensive parenchymal extinction and ductular reaction, secondary to alcohol abuse. mixed micro-macrovesicular steatosis involving about 50% of hepatocytes. |
| PHS19-24672 | 61 | M | Alcoholic hepatitis | mixed micro- and macronodular cirrhosis. clinical history of ethanol use.  areas of parenchymal extinction with marked ductular reaction replacement and focal cholestasis. |
| PHS19-31767 | 63 | M | Nonalcoholic steatohepatitis (NASH) | Mixed macro and micronodular cirrhosis clinically secondary to NASH (Nash activity score: 2/8) (fibrosis stage: 4/4). Areas of parenchymal extinction with marked ductular reaction replacement and  focal cholestasis. |
| PHS19-32169 | 66 | M | Alcoholic hepatitis | Macronodular cirrhosis, clinically due to Hepatitis C and alcoholic steatohepatitis. Focal areas of parenchymal extinction with marked ductular reaction  replacement. |
| PHS19-40351 | 42 | M | Alcoholic hepatitis | Predominantly micronodular cirrhosis secondary to chronic steatohepatitis occurring in setting of obesity and ethanol use. Areas of parenchymal extinction with marked ductular reaction. Hepatocyte ballooning with Mallory-Denk bodies, neutrophils and hepatocanalicular cholestasis. Macrovesicular steatosis involving approximately 30-40% of hepatocytes. |
| PHS20-9506 | 54 | F | NASH | Mixed micro- and macronodular cirrhosis; clinical non-alcoholic steatohepatitis. a. numerous alpha-1 antitrypsin globules in periportal hepatocytes highlighted by pas/d and AAT immunostain. Mild mixed steatosis involving approximately 5% of hepatocytes. Areas of parenchymal extinction with marked ductular reaction replacement. |
| PHS20-9600 | 56 | F | Alcoholic hepatitis | Mixed micro- and macronodular cirrhosis with minimal residual steatosis involving <5% of hepatocytes. Clinical history of non-alcoholic steatohepatitis. Areas of parenchymal extinction with marked ductular reaction replacement and focal cholestasis. |
| PHS20-10786 | 39 | F | Alcoholic hepatitis | Predominantly micronodular cirrhosis with large areas of parenchymal extension and florid ductular reaction. Clinically decompensated cirrhosis due to ethanol use. Focal ballooning degeneration with rare, poorly-formed, Mallory-Denk bodies and mega-mitochondria. |
| PHS16-44914 | 64 | F | Alcoholic hepatitis | Active mixed cirrhosis with focal parenchymal extinction. Focally severe mixed micro-macrovesicular steatosis involving 60-70% of hepatocytes with superimposed steatohepatitis, easily identifiable Mallory-Denk bodies and cholangiolar cholestasis. Consistent with clinical history of alcohol use. |
| PHS17-14821 | 62 | M | Alcoholic hepatitis | Active mixed but predominantly micronodular cirrhosis with residual mixed steatosis and occasional Mallory's hyaline deposition. Clinical history of alcohol-use. Occasional regenerative nodules with brisk ductular reaction. |
| PHS16-28155 | 67 | F | Polycystic liver disease | Benign cysts lined by biliary epithelium. Surrounding liver parenchyma with chronic inflammation, patchy scarring and multiple biliary hamartomas (Von Meyenberg complexes). Mild macrovesicular steatosis involving about 10% of hepatocytes. Findings consistent with clinical history of polycystic liver and kidney disease. |
| PHS12-30089 | 60 | F | Polycystic liver disease | Multiple biliary type cysts, consistent with the clinical history of polycystic liver  disease. Vascular congestion and mild chronic inflammation of the cyst wall. Small islands of hepatocytes with sinusoidal congestion and mild microvesicular steatosis |
| PHS15-21076 | 35 | F | Polycystic liver disease | Polycystic liver disease with Von Meyenberg complexes. Nodular regenerative hyperplasia and mild non-specific portal as well as lobular inflammation. |
| PHS12-35452 | 50 | F | Polycystic liver disease | Multiple biliary cysts, consistent with polycystic liver disease. Extensive hemorrhage, chronic inflammation, and fibrosis in the wall of biliary cysts. |
| PHS10-4073 | 48 | F | Polycystic liver disease | Multiple biliary cysts and multiple Von Meyenburg's complexes, consistent with the clinical history of adult polycystic kidney and liver disease. |
| PHS12-34148 | 34 | F | Cystic fibrosis | Bile ductular proliferation with cholangiolitis. Portal fibrosis with focal portal to portal early fibrous bridge formation. Histologic changes are nonspecific and are compatible with the underlying diagnosis of cystic fibrosis. |
| PHS15-14513 | 32 | M | Cystic fibrosis | Large areas of fibrosis with thick fibrous bands suggestive of marked architectural  distortion. Numerous occasional hepatocytes with pseudo-ground glass cytoplasm. Architectural distortion with fibrous bands. The clinical history of lung transplantation secondary to cystic fibrosis is noted. The pattern of fibrosis development in patients with cystic fibrosis can be irregular and focal, leading to incomplete hepatic fibrosis (focal biliary cirrhosis/fibrosis). |
| PHS16-1251 | 23 | F | Cystic fibrosis | Intact hepatic architecture with diffusely increased hepatocyte glycogen. Mild diminution in portal vein caliber. Mild microvesicular steatosis. Mild increase in reticuloendothelial iron stores History of elevated alkaline phosphatase, AST, GGTP and ammonia with normal bilirubin and ALT levels who underwent double lung transplant in november 2015 for treatment of cystic fibrosis. |
| PHS17-19070 | 23 | M | Cystic fibrosis | Prominent macrovesicular steatosis involving approximately 90% of sampled liver parenchyma with superimposed minimally active steatohepatitis (NAS active score = 4-5/8). Portal, periportal and pericellular fibrosis with scattered delicate non-bridging fibrous septae (fibrosis stage = 2-3/4). Focal marked ductular proliferation with associated cholangiolitis, ductular ectasia and inspissated luminal secretions. Findings consistent with the clinical history of cystic fibrosis |
| PHS17-35744 | 22 | M | Cystic fibrosis | Cystic fibrosis related liver disease with PAS-positive inspissated bile plugs and patchy periportal/sinusoidal fibrosis (fibrosis stage 2/4). Bile duct dilatation with cholangiolar proliferation, neutrophilic cholangitis, lobular ballooning degeneration and canalicular cholestasis. Consistent with cystic fibrosis. |
| TP10-P531 | 31 | F | Cystic fibrosis | Decompensated cirrhosis in the setting of cystic fibrosis. Cholangiolitis with marked ductular proliferation, ductular ectasia and biliary sludge consistent with CF. Severe mixed micro- and macro-vesicular steatosis. NAS=6/8 and fibrosis stage=4/4 |
